# What killed Frame Lake? A precautionary tale for urban planners

**DOI:** 10.7717/peerj.4850

**Published:** 2018-06-14

**Authors:** Melody J. Gavel, R. Timothy Patterson, Nawaf A. Nasser, Jennifer M. Galloway, Bruce W. Hanna, Peter A. Cott, Helen M. Roe, Hendrik Falck

**Affiliations:** 1 Department of Earth Sciences and Ottawa-Carleton Geoscience Centre, Carleton University, Ottawa, Ontario, Canada; 2 Geological Survey of Canada Calgary/Commission Géologique du Canada, Calgary, Alberta, Canada; 3 GNWT-WLU, Wilfrid Laurier University, Waterloo, Ontario, Canada; 4 Cott Environmental, Yellowknife, Northwest Territories, Canada; 5 School of Natural and Built Environment, Queen’s University, Belfast, United Kingdom; 6 Northwest Territories Geological Survey, Yellowknife, Northwest Territories, Canada

**Keywords:** Eutrophication, Urbanization, Arsenic contamination, Frame lake, Arcellinida

## Abstract

Frame Lake, located within the city of Yellowknife, Northwest Territories, Canada, has been identified as requiring significant remediation due to its steadily declining water quality and inability to support fish by the 1970s. Former gold mining operations and urbanization around the lake have been suspected as probable causes for the decline in water quality. While these land-use activities are well documented, little information is available regarding their impact on the lake itself. For this reason, Arcellinida, a group of shelled protozoans known to be reliable bioindicators of land-use change, were used to develop a hydroecological history of the lake. The purpose of this study was to use Arcellinida to: (1) document the contamination history of the lake, particularly related to arsenic (As) associated with aerial deposition from mine roaster stacks; (2) track the progress of water quality deterioration in Frame Lake related to mining, urbanization and other activities; and (3) identify any evidence of natural remediation within the lake. Arcellinida assemblages were assessed at 1-cm intervals through the upper 30 cm of a freeze core obtained from Frame Lake. The assemblages were statistically compared to geochemical and loss-on-ignition results from the core to document the contamination and degradation of conditions in the lake. The chronology of limnological changes recorded in the lake sediments were derived from ^210^Pb, ^14^C dating and known stratigraphic events. The progress of urbanization near the lake was tracked using aerial photography. Using Spearman correlations, the five most significant environmental variables impacting Arcellinida distribution were identified as minerogenics, organics, As, iron and mercury (*p* < 0.05; *n* = 30). Based on CONISS and ANOSIM analysis, three Arcellinida assemblages are identified. These include the Baseline Limnological Conditions Assemblage (BLCA), ranging from 17–30 cm and deposited in the early Holocene >7,000 years before present; the As Contamination Assemblage (ACA), ranging from 7–16 cm, deposited after ∼1962 when sedimentation began in the lake again following a long hiatus that spanned to the early Holocene; and the Eutrophication Assemblage (EA), ranging from 1–6 cm, comprised of sediments deposited after 1990 following the cessation of As and other metal contaminations. The EA developed in response to nutrient-rich waters entering the lake derived from the urbanization of the lake catchment and a reduction in lake circulation associated with the development at the lake outlet of a major road, later replaced by a causeway with rarely open sluiceways. The eutrophic condition currently charactering the lake—as evidenced by a population explosion of eutrophication indicator taxa *Cucurbitella tricuspis*—likely led to a massive increase in macrophyte growth and winter fish-kills. This ecological shift ultimately led to a system dominated by *Hirudinea* (leeches) and cessation of the lake as a recreational area.

## Introduction

The City of Yellowknife, located in the Northwest Territories (NWT), Canada, is near to several former gold mining operations, most notably the Giant Mine (1948–2004)and Con Mine (1938–2003), which have been identified as requiring considerable remediation to contain legacy contamination (GNWT-ITI, 2007; Government of Canada, 2013a). In particular, mineral processing activities in the early years of operation resulted in the contamination of the surrounding landscape, including lakes and streams, by arsenic trioxide (As_2_O_3_) and other metals of concern (e.g., zinc, lead, and iron) (DIAND, 2007). One lake that has elicited particular interest is the heavily impacted Frame Lake, located within the Yellowknife city limits (Government of Canada, 2013a). Frame Lake serves as an important recreational and meeting area for both tourists and local citizens, with a walking trail extending around the periphery, and with park lands, Yellowknife City Hall, the Legislative Assembly of the Northwest Territories, and the Prince of Wales Northern Heritage Center located on its shore. Historically the lake, originally known as *Enaàtì*, was the site of a fishing camp used by the Yellowknives Dene First Nations, where Lake Whitefish (*Coregonus clupeaformis* Mitchell 1818), Northern Pike (*Esox lucius* Linnaeus 1758), and suckers (*Catostomus* spp.) were harvested and dried in the fall for use in the winter. The camp was abandoned in the 1930s when industrial activity commenced in the area (Yellowknives Dene First Nations, 2016). Though the lake was once popular for swimming at McNiven Beach (Fig. S1), and fishing through the 1950s and 1960s, the disappearance of fish from the lake prior to the 1970s, with concurrent increase in dense macrophyte growth, as well as an increase in leech (*Hirudinea*) populations, eventually led to a cessation in recreational use of its waters. Though the exact date of the disappearance of fish is uncertain, a 1973 study did not observe any fish in the lake (Healey & Woodall, 1973). It has been hypothesized that the disappearance of fish from the lake was a result of steady water quality deterioration from areal deposition from mining processes, use of the lake as a winter snow dump, urbanization of the area around the lake that disrupted lake circulation, and possible unregulated dumping of sewage, mine and other waste. Reports of dumping in the lake are controversial, as there is no direct written record of such occurrences. However, some long-time Yellowknife citizens claim to have witnessed such incidents first hand (V Sterenberg, former Yellowknife mayors, D Lovell & G Van Tighem, pers. comm., 2015; Patterson et al., 2015a).

When detailed records of historical land use are absent, biological proxies can be used to track changes in ecology. Arcellinida (also known as testate lobose amoebae or thecamoebians) are a group of unicellular protozoans that are found worldwide in freshwater or brackish environments (Medioli & Scott, 1983; Medioli & Scott, 1988; Patterson & Kumar, 2000a; Patterson & Kumar, 2000b). Due to arcellinidans having a rapid reproduction rate and demonstrated high down-core preservation potential, they have become a useful tool in monitoring the health of lacustrine environments (Patterson & Kumar, 2000a; Patterson & Kumar, 2000b). In particular, their rapid response to contamination events makes arcellinidans an important tool in lacustrine bio-monitoring and remediation studies (Patterson, Barker & Burbidge, 1996; Reinhardt et al., 2005; Patterson et al., 2013). Previous research using Arcellinida as proxies of mining contamination in Yellowknife area, NWT surface samples has shown that the group is responsive to arsenic contamination (Nasser et al., 2016). This makes Arcellinida an ideal proxy for documenting the hydroecological and geochemical changes that occurred in Frame Lake in response to contamination.

There have only been a few previous studies carried out in Frame Lake. Healey & Woodall (1973) provided documentation that by the time of their study the lake no longer supported a fish population and suggested that this was the result of urbanization-induced eutrophication. They proposed that eutrophication led to excessive macrophyte growth, which in turn led to reduced oxygen levels as the vegetable matter decomposed during the winter leading to fish winterkill. Dirszowsky et al. (2013) reported that Frame Lake had become increasingly eutrophic through the last 80 years and that the lake sediments also contained elevated levels of contaminants including arsenic, lead, and zinc. Dirszowsky & Wilson (2016) also documented high concentrations of arsenic, which they attributed to being derived from historical mining activities. They observed that there had been an increase in nutrification within Frame Lake, and concluded that the lake was particularly sensitive to nutrient loading. Other research conducted on sediment/water interface samples lakes of the Yellowknife region has suggested that the high levels of arsenic observed were a legacy of air fall from roaster stacks associated with area mining operations (Galloway et al., 2010; Galloway et al., 2017). Studies conducted on the benthic invertebrate communities and macrophytes found in the lake have provided further verification of the eutrophic and contaminated conditions of the lake (Moore, 1978; Koch et al., 2000). However, aside from one study, focused on diatoms, that documented the progressively eutrophic nature of the lake in recent decades (Dirszowsky et al., 2013), there have been no temporal studies conducted on lake sediments to track changes in biological communities in response to climate change or anthropogenic impacts on the lake.

The purpose of this study is to: (1) track the hydroecological changes that have occurred in Frame Lake in response to mining contamination, urbanization, and possible illegal dumping; and (2) track the progress of any possible lake remediation that may have occurred since the cessation of contaminants derived from air fall, illegal dumping and other sources related to urbanization around the lake, and (3) provide further support for the use of Arcellinida as bio-indicators of arsenic contamination.

### Study area

**Figure 1:**
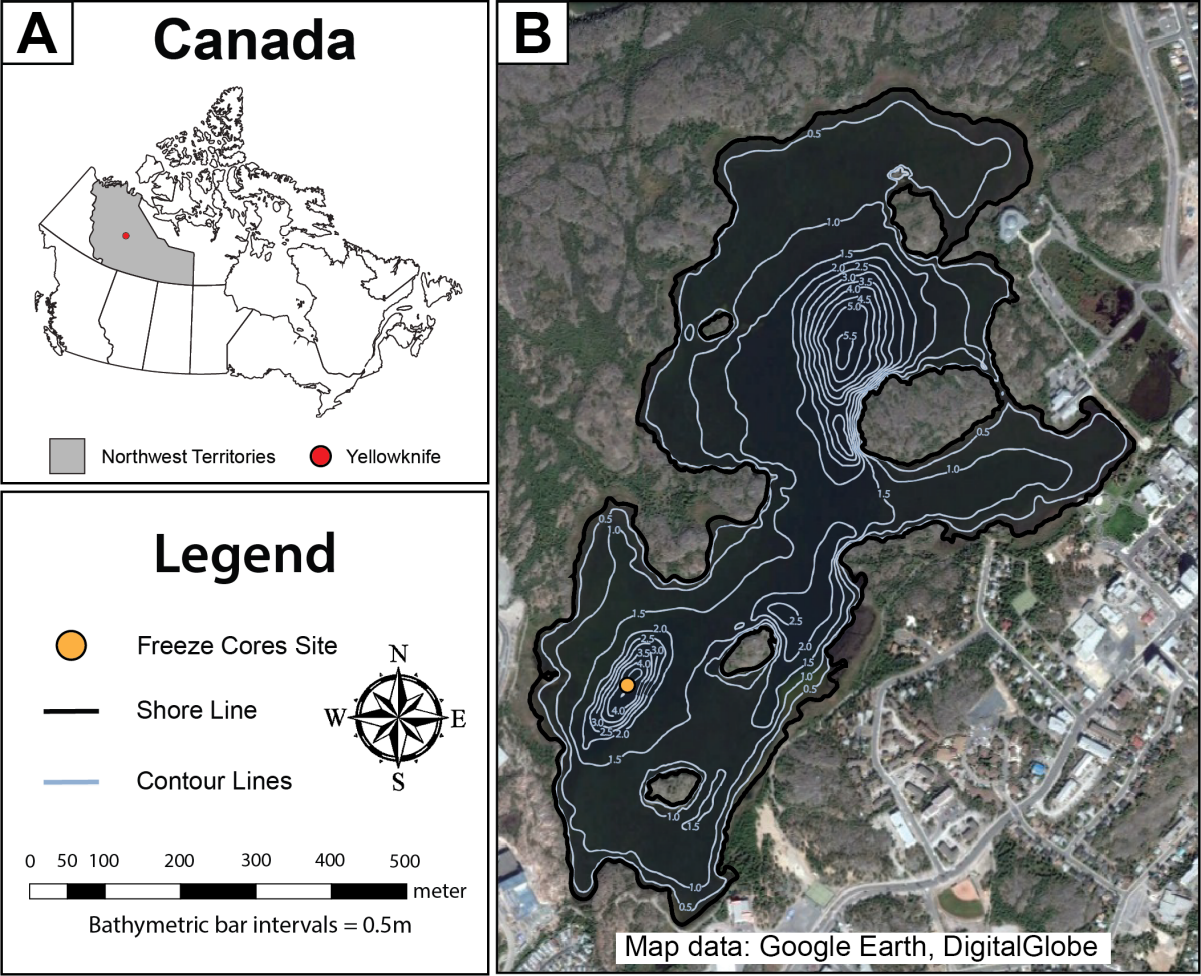
Map of Frame Lake. (A) a map showing the location of the study area in Canada, and (B) an aerial photo showing Frame Lake. The freeze coring site is represented by a yellow circle, while the lake outlines and bathymetry contours are represented by the black and light blue lines, respectively (Map data: Google Earth, DigitalGlobe).

Frame Lake is located adjacent to the downtown area of the City of Yellowknife (Fig. 1). The lake is eutrophic, and has arguably become endorheic in recent decades, due to disruption of inflow and outflow streams as a result of urban land use change. The lake has a maximum depth of 4.5 m in the Southern Basin and 5.5 m in the northern basin. The Yellowknife area experiences about 100 frost-free days a year, with ice normally covering Frame Lake by the end of October through late May or early June. The maximum depth of ice-cover in small Yellowknife area lakes, including Frame Lake, is approximately 1.0 m (Dirszowsky et al., 2013). The lake is dominated by extensive macrophyte cover, reflecting its highly nutrified state (Fig. S2). The landscape of the Yellowknife area can be described as a low relief (10–20 m) rolling plain, underlain by Canadian Shield geology. Drainage is influenced by this underlying bedrock, with most lakes draining into Great Slave Lake (Dirszowsky et al., 2013).

### Anthropogenic impact

**Figure 2:**
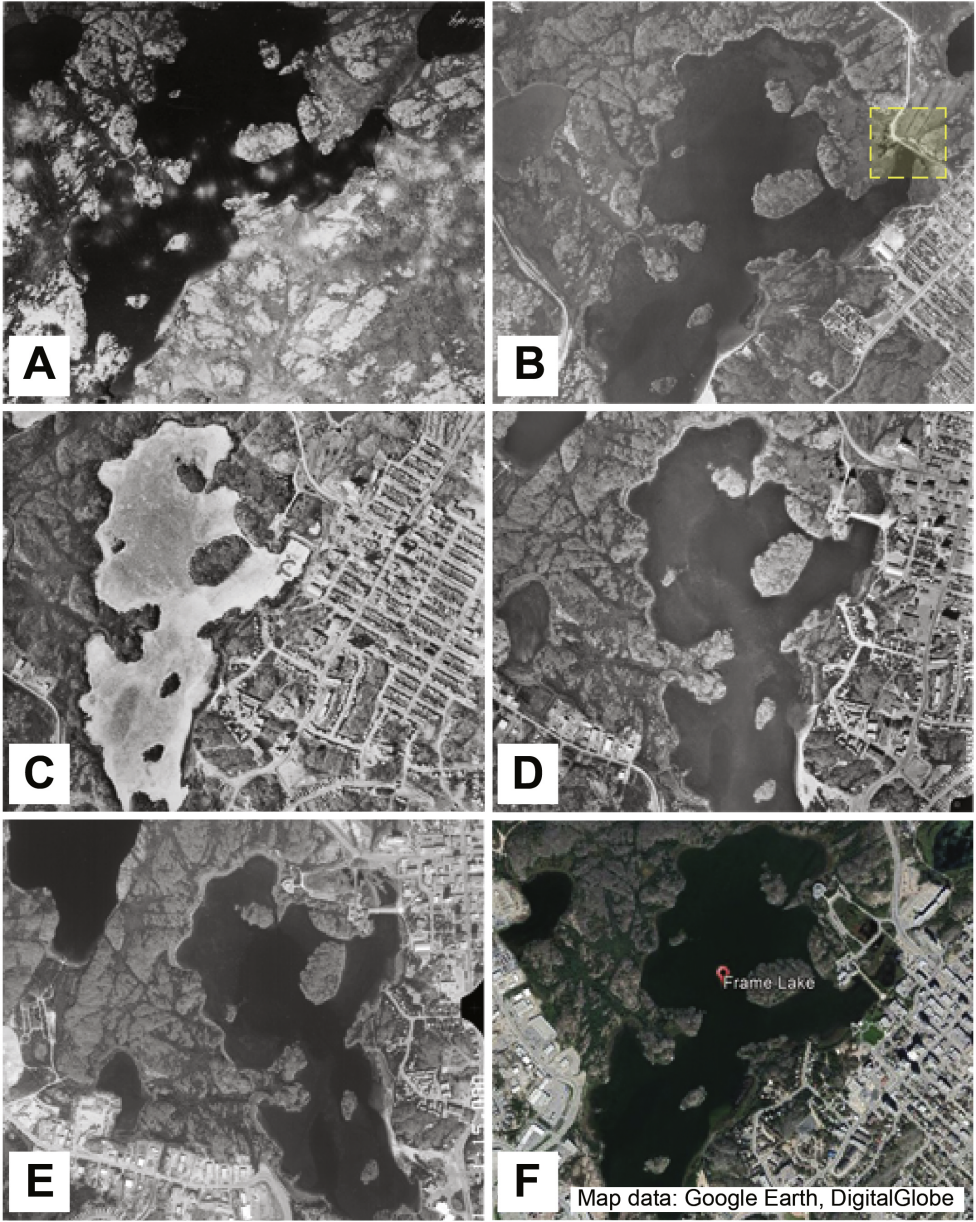
Aerial photos tracking 80 years of development around Frame Lake. (A) Frame Lake in 1937 (A5611-049, NAPL 2017). (B) Frame Lake in 1964 (A18310-054, NAPL 2017), with a yellow box indicating the construction of a major road across a portion of the lake. (C) Frame Lake in 1976 (A24380-097). (D) Frame Lake in 1985 (A26715-215). (E) Frame Lake in 1996 (A28280-111). (F) Frame Lake in 2017 (Map data: Google Earth, DigitalGlobe).

The development that would eventually result in the founding of the City of Yellowknife is linked to the discovery of gold in the area in 1896 (Moir et al., 2006; Government of Canada, 2013b). The opening of three major gold mines (Giant Mine, Con Mine and Discovery Mine) in the 1930s led to the rapid expansion and urbanization of Yellowknife through the subsequent decades, with major growth occurring after the city was established as the territorial capital in 1967 (Fig. 2) (Moir et al., 2006). The world class Giant Mine went on to be the most successful mine in the area, producing 7.6 million ounces of gold from the time of first production in 1948 to its closure in 2004. The separation of ore from gold at the Giant Mine was also responsible for the production of 237,000 tonnes of arsenic trioxide (As_2_O_3_) waste as a by-product of the ore-roasting process (GNWT-RWED, 1993). In the early days of the Giant Mine, a lax regulatory environment resulted in the direct release of As_2_O_3_ dust into the atmosphere via stack emissions. Although the Giant Mine operators eventually implemented more sophisticated technologies to reduce arsenic aerial emissions by the 1950s, methods which improved over time, approximately 19 million kg of As_2_O_3_ was released into the atmosphere. These aerial emissions fell on the surrounding landscape, which served as a major contaminant legacy in the area (Palmer et al., 2015; Galloway et al., 2010; Galloway et al., 2017; Government of Canada, 2013b).

The City of Yellowknife eventually grew to encompass the area surrounding Frame Lake. Due to its centralized location within the city and low inflow/outflow rates, the lake is susceptible to sediment loading. Water quality in the lake has thus been hypothesized to have not only been negatively impacted by (1) As_2_O_3_ originating from aerial mining emissions, but also by (2) the construction of a causeway in the mid-1970s that disrupted outflow, and (3) the construction of roads and built infrastructure in the 1960s and 1970s that disrupted inflow to the lake, and (4) surface runoff of degraded water quality from urbanization around the lake (Fig. 2). There are anecdotal reports of the physical dumping of mine waste and sewage into the lake, and there is documentation that the lake was used as a snow dump, which would have contained a considerable sediment content that would have been scraped up by the plows (D Lovell, pers. comm., 2015). These sediment sources would have contributed to both an increase in sedimentation rate in the lake and would have contributed to the deterioration of the lake’s water quality. An increase in extensive lake bottom vegetation, which upon dying down each winter, has also contributed to an increase in sedimentation in the lake (Dirszowsky et al., 2013; Patterson et al., 2015a). This increase in sedimentation, coupled with low turnover rates and contamination, eventually led to the significant nutrification of Frame Lake and development of winter dysoxic conditions (Dirszowsky et al., 2013; Patterson et al., 2015a).

## Materials and Methods

### Field work and sampling design

**Figure 3:**
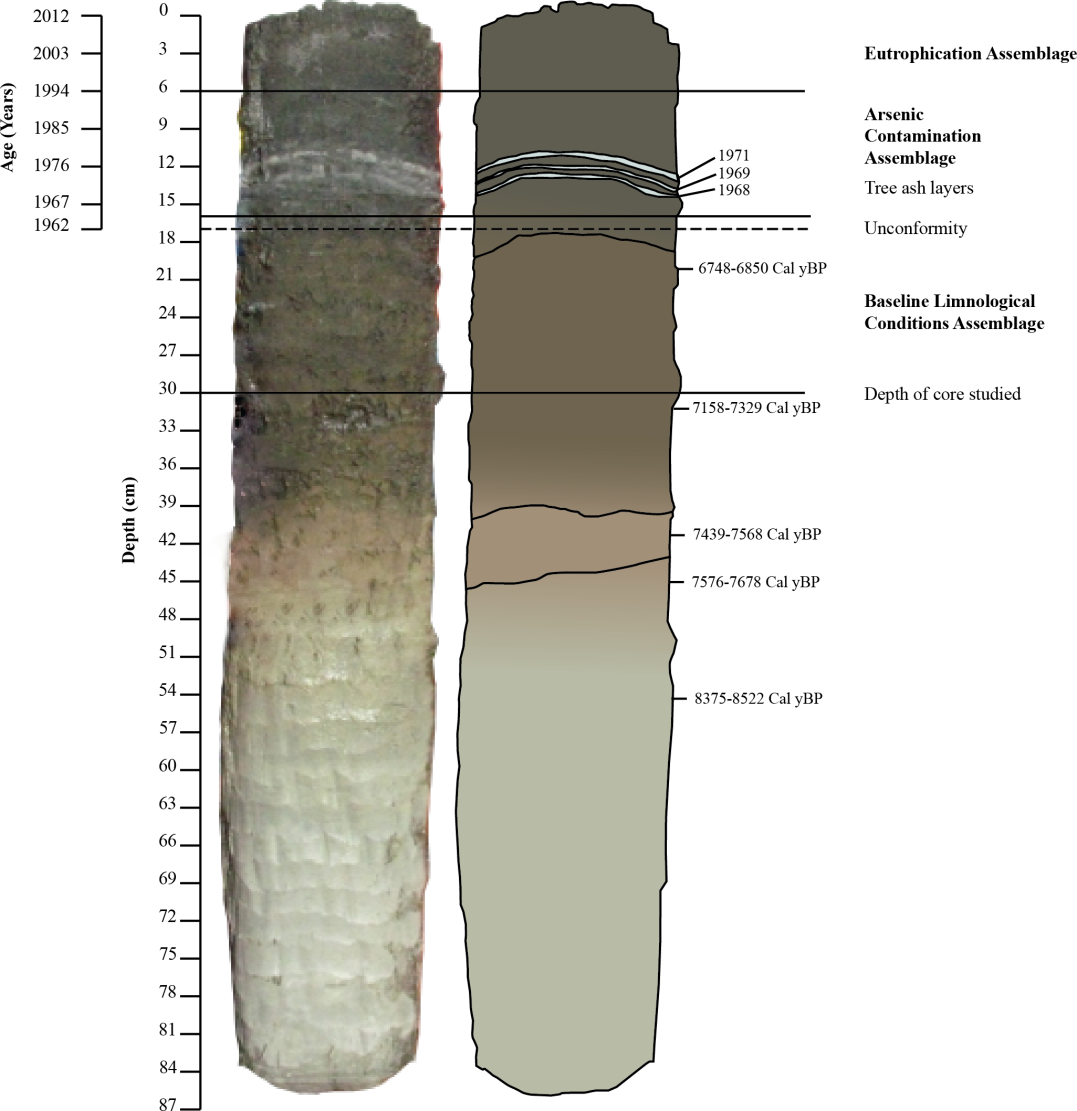
Freeze core 1FR with Pb^210^, ash layer, and radiocarbon dates. Pb^210^ dates for the upper 17 cm of the core as well as ash layer dates (D Lovell, pers. comm., 2015), radiocarbon dates from 18 to 30 cm; ash layers, assemblages and location of unconformity.

The field work component of this study was carried out under the Aurora Research Institute licence nos. 14435 and 14965. Three sediment cores were retrieved from the southern basin of Frame Lake using a two-faced rectangular freeze corer and a single faced freeze corer in September 2012 (Fig. 3). The two cores obtained using the two-faced freeze corer were from a depth of ∼4.6 m and were 60.0 cm (core 2FRF1) and 60.4 cm in length (core 2FRF2). The core obtained using the singe-faced corer (1FR) from a water depth of 4.1 m was 86 cm long (see Galloway et al., 2010 for detailed description of core collection method). The freeze cores were shipped to Carleton University for further analysis. The stratigraphy of the cores was logged at Carleton University, and color variation was documented using a Munsell colour chart (Munsell Color Company, Inc, 2000).

### Laboratory analyses and chronology

The freeze cores used in this research were subsampled for the various proxy analyses using a custom designed sledge microtome (Macumber et al., 2011) at sampling intervals ranging from 0.1 to 0.5 cm. The single faced core (1FR) was sliced at a resolution varying between 0.2 and 0.5 cm to a depth of 60 cm and the samples were commercially analyzed by ACME Analytical Laboratories, Vancouver, using inductively coupled mass spectrometry (ICP-MS) following Aqua Regia digestion (ICP-MS 1F/AQ250 package) to detect environmentally important metals that are bioavailable (EPA, 2011) and because complete digestion methods, for example multi-acid digestion, can volatilize As, a contaminant of potential concern in this study (Table S1) (Parsons et al., 2012).

Core 2FRF1 was sliced to 0.5 cm resolution to a depth of 15 cm and submitted for radiocarbon dating to the André E Lalonde (AEL) AMS laboratories (AMS, 2014). Additional × range finder radiocarbon dates were obtained from lower in the core. Chronology for the 20th and 21st century uppermost part of the core was based on ^210^Pb dates obtained from a fourth Frame Lake core collected from a few meters away in the same basin in 2014, as well as from three ash layers derived from New Year’s Christmas tree bonfires that were held on the lake in 1968, 1969 and 1971 (V Sterenberg, former Yellowknife Mayor, D Lovell, pers. comm., 2015) (Fig. 3).

Core 2FRF2 was sliced to 0.1 cm resolution for various proxy analyses to a depth of 30 cm. Arcellinida analysis was carried out on subsamples at 1 cm resolution. Three cc’s of wet material from the Arcellinidan aliquot were sieved first at 297 µm then at 37 µm to remove both coarse and fine organic and mineral debris. The samples were then split into six aliquots for micropaleontological analysis using a wet splitter (Scott & Hermelin, 1993). Arcellinida in each sample were quantified using an Olympus SZH dissecting binocular microscope (Olympus, Center Valley, PA, USA) at magnifications ranging between 20 and 50× until counts of at least 150 were obtained. Total carbonates and organic carbon were measured at 1 cm intervals in the 0.1 cm interval immediately adjacent to those used for micropaleontological analysis using loss on ignition (LOI) analysis (Ball, 1964).

### Aerial photography

Aerial photos documenting the urbanization around Frame Lake between 1937 and 1996 were acquired through the National Air Photo Library (NAPL)—Natural Resources Canada. Air photos from 1937, 1964, 1976, 1985 and 1996 were used to show major development around the lake (Fig. 2) Google Earth was used to acquire a 2017 image of Frame Lake (Fig. 2).

### Statistical analysis

Thirty-two Arcellinidan species and strains were identified using several well-illustrated papers that utilized the strain taxonomic concept as identification guides (e.g., Reinhardt et al., 1998; Patterson et al., 2013; Patterson et al., 2015b; Nasser et al., 2016) in 30 samples and converted to relative abundances (Table 1). For illustrated specimens of the arcellinidan pertaining to local NWT Arcellinida, refer to Nasser et al. (2016). Probable error (*pe*) was calculated for each sample using the formula:

\begin{eqnarray*}& & pe=1.96 \left( \frac{s}{\sqrt{{X}_{i}}} \right) \end{eqnarray*}

where *s* is the standard deviation of the population and *X*_*i*_ is the fractional abundance (Patterson & Fishbein, 1989). A sample was considered statistically significant if the total counts in the sample were greater than the probable error. All 30 samples were deemed statistically significant. The following formula was used to calculate standard error (*S*_*Xi*_) for each species:

\begin{eqnarray*}& & {S}_{Xi}=1.96\sqrt{ \frac{{X}_{i}(1-{X}_{i})}{{N}_{i}} } \end{eqnarray*}

where *X*_*i*_ is fractional abundance of each species and *N*_*i*_ is the total species counts in a sample (Patterson & Fishbein, 1989). Species were included if their standard error was smaller than the fractional abundance. Based on these calculations, 32 species were deemed statistically significant and included in the calculations.

**Table 1:** Shannon diversity index values, difflugid/centropyxid ratios, loss-on-ignition results, and relative abundances of arcellinidan counts.

Sample	1	2	3	4	5	6	7	8	9	10	11	12	13	14	15	16	17	18	19	20	21	22	23	24	25	26	27	28	29	30
Shannon index (H)	2.41	2.14	2.47	2.49	2.65	2.36	2.09	2.38	2.37	2.20	2.47	2.27	2.06	2.41	2.45	2.60	2.61	2.59	2.45	2.38	2.48	2.36	2.26	2.39	2.19	2.37	2.42	2.57	2.35	2.27
Difflugid/ centropyxid ratio	0.70	0.70	0.55	0.69	0.66	0.63	0.20	0.21	0.26	0.30	0.33	0.32	0.28	0.45	0.40	0.54	0.62	0.68	0.57	0.66	0.80	0.85	0.89	0.90	0.92	0.86	0.77	0.75	0.80	0.82
Water	0.01	0.03	0.03	0.05	0.04	0.05	0.06	0.06	0.08	0.12	0.18	0.16	0.16	0.16	0.12	0.09	0.08	0.11	0.10	0.10	0.08	0.08	0.09	0.09	0.10	0.10	0.10	0.12	0.09	0.09
Organics	0.35	0.36	0.31	0.34	0.34	0.40	0.34	0.32	0.21	0.20	0.10	0.12	0.15	0.15	0.18	0.24	0.33	0.28	0.32	0.35	0.37	0.35	0.35	0.35	0.35	0.36	0.35	0.38	0.35	0.33
Carbonates	0.03	0.02	0.03	0.01	0.02	0.03	0.02	0.02	0.03	0.02	0.02	0.02	0.02	0.02	0.02	0.03	0.03	0.02	0.02	0.02	0.02	0.03	0.03	0.02	0.02	0.02	0.02	0.02	0.02	0.02
Minerogenics	0.63	0.62	0.66	0.65	0.64	0.57	0.64	0.66	0.76	0.79	0.88	0.86	0.83	0.83	0.80	0.73	0.64	0.70	0.66	0.63	0.61	0.63	0.62	0.63	0.63	0.62	0.63	0.60	0.63	0.64
*Arcella vulgaris*	0.00	0.00	0.00	0.00	0.00	0.00	0.02	0.00	0.00	0.01	0.01	0.01	0.00	0.00	0.00	0.00	0.00	0.00	0.00	0.00	0.00	0.00	0.00	0.00	0.00	0.00	0.00	0.00	0.01	0.01
*Centropyxis aculeata “aculeata”*	0.01	0.03	0.01	0.04	0.03	0.05	0.13	0.14	0.14	0.10	0.17	0.07	0.07	0.07	0.07	0.03	0.11	0.06	0.14	0.13	0.06	0.03	0.00	0.01	0.00	0.02	0.07	0.08	0.06	0.09
*Centropyxis aculeata “discoides”*	0.01	0.03	0.04	0.04	0.05	0.03	0.18	0.14	0.12	0.06	0.10	0.07	0.06	0.05	0.06	0.05	0.09	0.09	0.05	0.03	0.01	0.02	0.01	0.00	0.00	0.00	0.03	0.06	0.02	0.02
*Centropyxis constricta “aerophila”*	0.15	0.08	0.23	0.10	0.13	0.13	0.23	0.23	0.27	0.17	0.17	0.26	0.27	0.21	0.29	0.20	0.11	0.10	0.14	0.07	0.03	0.03	0.03	0.04	0.02	0.05	0.07	0.01	0.02	0.01
*Centropyxis constricta “constricta”*	0.04	0.02	0.05	0.04	0.07	0.07	0.10	0.14	0.14	0.19	0.16	0.23	0.28	0.18	0.16	0.15	0.04	0.06	0.08	0.09	0.08	0.07	0.06	0.04	0.05	0.05	0.04	0.07	0.07	0.05
*Centropyxis constricta “spinosa”*	0.00	0.00	0.01	0.00	0.00	0.00	0.00	0.02	0.02	0.01	0.00	0.00	0.00	0.00	0.01	0.02	0.02	0.01	0.01	0.01	0.01	0.00	0.00	0.00	0.01	0.01	0.01	0.01	0.01	0.00
*Centropyxis pontigulasiformis*	0.00	0.00	0.00	0.00	0.01	0.00	0.00	0.00	0.00	0.00	0.00	0.00	0.00	0.00	0.00	0.00	0.00	0.00	0.00	0.00	0.00	0.00	0.00	0.00	0.00	0.00	0.00	0.00	0.00	0.00
*Cucurbitella tricuspis*	0.19	0.39	0.18	0.22	0.15	0.19	0.18	0.10	0.03	0.18	0.06	0.05	0.04	0.04	0.03	0.04	0.01	0.01	0.01	0.00	0.02	0.01	0.03	0.01	0.05	0.01	0.01	0.01	0.03	0.03
*Difflugia bidens*	0.00	0.00	0.01	0.01	0.03	0.01	0.00	0.00	0.00	0.00	0.01	0.03	0.00	0.00	0.01	0.01	0.00	0.00	0.01	0.00	0.00	0.00	0.01	0.01	0.01	0.00	0.00	0.01	0.00	0.00
*Difflugia urceolata “urceolata”*	0.00	0.00	0.00	0.00	0.00	0.00	0.00	0.00	0.01	0.00	0.01	0.01	0.03	0.02	0.00	0.03	0.01	0.00	0.01	0.00	0.01	0.01	0.00	0.02	0.01	0.00	0.01	0.03	0.01	0.05
*Difflugia urceolata “elongata”*	0.00	0.00	0.01	0.01	0.01	0.07	0.02	0.00	0.01	0.04	0.01	0.02	0.01	0.04	0.02	0.04	0.03	0.07	0.03	0.05	0.04	0.03	0.02	0.05	0.03	0.03	0.05	0.05	0.00	0.03
*Difflugia urens*	0.01	0.00	0.00	0.01	0.05	0.00	0.00	0.00	0.03	0.00	0.02	0.00	0.00	0.01	0.02	0.02	0.03	0.02	0.00	0.02	0.05	0.03	0.00	0.01	0.00	0.00	0.01	0.01	0.03	0.00
*Lesquereusia spiralis*	0.02	0.02	0.01	0.03	0.00	0.02	0.00	0.04	0.01	0.00	0.02	0.01	0.01	0.01	0.00	0.01	0.00	0.00	0.00	0.01	0.00	0.00	0.01	0.00	0.00	0.00	0.00	0.00	0.00	0.00
*Lagenodifflugia vas*	0.03	0.00	0.01	0.01	0.01	0.00	0.00	0.01	0.01	0.00	0.01	0.01	0.00	0.01	0.00	0.00	0.02	0.02	0.02	0.01	0.03	0.04	0.03	0.05	0.03	0.04	0.03	0.01	0.02	0.01
*Pontigulasia compressa*	0.01	0.00	0.01	0.00	0.00	0.00	0.00	0.01	0.00	0.00	0.00	0.00	0.00	0.01	0.00	0.00	0.01	0.00	0.00	0.01	0.01	0.00	0.00	0.01	0.01	0.00	0.00	0.01	0.02	0.01
*Difflugia glans “magna”*	0.00	0.00	0.00	0.00	0.01	0.00	0.00	0.01	0.00	0.00	0.00	0.00	0.00	0.00	0.00	0.00	0.00	0.00	0.00	0.00	0.00	0.00	0.00	0.00	0.00	0.00	0.00	0.01	0.00	0.00
*Difflugia glans “distenda”*	0.00	0.01	0.00	0.01	0.01	0.00	0.00	0.00	0.00	0.00	0.00	0.00	0.00	0.00	0.02	0.00	0.01	0.03	0.03	0.01	0.05	0.01	0.00	0.00	0.00	0.00	0.00	0.00	0.00	0.00
*Medilous corona*	0.04	0.02	0.02	0.03	0.00	0.01	0.00	0.01	0.02	0.04	0.01	0.01	0.00	0.01	0.00	0.01	0.00	0.00	0.00	0.00	0.00	0.00	0.03	0.04	0.01	0.01	0.01	0.01	0.00	0.01
*Difflugia oblonga “oblonga”*	0.10	0.11	0.07	0.10	0.03	0.04	0.04	0.04	0.01	0.10	0.05	0.07	0.05	0.09	0.05	0.09	0.20	0.19	0.22	0.25	0.05	0.11	0.28	0.18	0.23	0.17	0.17	0.13	0.27	0.24
*Difflugia oblonga “spinosa”*	0.15	0.11	0.11	0.19	0.02	0.26	0.07	0.02	0.01	0.00	0.00	0.00	0.00	0.00	0.01	0.03	0.06	0.02	0.01	0.01	0.01	0.03	0.07	0.13	0.11	0.15	0.11	0.14	0.11	0.09
*Difflugia oblonga “lanceolata”*	0.00	0.00	0.00	0.00	0.00	0.00	0.00	0.00	0.00	0.00	0.00	0.00	0.00	0.00	0.02	0.01	0.00	0.00	0.00	0.00	0.00	0.00	0.00	0.00	0.00	0.01	0.01	0.01	0.00	0.00
*Difflugia oblonga “tenuis”*	0.03	0.07	0.04	0.00	0.08	0.03	0.01	0.01	0.00	0.00	0.00	0.01	0.01	0.01	0.03	0.02	0.05	0.02	0.01	0.03	0.21	0.16	0.03	0.05	0.03	0.03	0.03	0.02	0.02	0.01
*Difflugia oblonga “glans”*	0.04	0.01	0.01	0.00	0.12	0.01	0.00	0.06	0.07	0.00	0.04	0.09	0.09	0.09	0.03	0.06	0.03	0.03	0.05	0.05	0.03	0.05	0.00	0.00	0.05	0.01	0.04	0.02	0.03	0.02
*Difflugia oblonga “triangularis”*	0.00	0.00	0.00	0.01	0.01	0.01	0.00	0.00	0.00	0.00	0.00	0.00	0.00	0.00	0.00	0.00	0.00	0.00	0.00	0.00	0.00	0.00	0.00	0.00	0.00	0.00	0.00	0.00	0.00	0.00
*Difflugia oblonga “linearis”*	0.00	0.00	0.00	0.00	0.00	0.01	0.00	0.00	0.00	0.00	0.00	0.00	0.00	0.00	0.01	0.00	0.00	0.00	0.00	0.01	0.00	0.00	0.01	0.01	0.01	0.01	0.00	0.00	0.00	0.00
*Difflugia protaeiformis “protaeiformis”*	0.00	0.01	0.01	0.00	0.00	0.01	0.01	0.01	0.01	0.00	0.01	0.00	0.01	0.01	0.01	0.01	0.03	0.04	0.05	0.01	0.04	0.01	0.01	0.03	0.00	0.02	0.00	0.01	0.02	0.05
*Difflugia protaeiformis “claviformis”*	0.00	0.01	0.09	0.01	0.07	0.01	0.01	0.00	0.01	0.00	0.06	0.03	0.04	0.06	0.07	0.11	0.03	0.07	0.04	0.06	0.01	0.05	0.05	0.03	0.05	0.09	0.02	0.04	0.06	0.02
*Difflugia protaeiformis “curvicaulis”*	0.00	0.02	0.02	0.00	0.01	0.00	0.00	0.00	0.01	0.00	0.01	0.00	0.00	0.00	0.01	0.01	0.01	0.01	0.00	0.00	0.00	0.00	0.01	0.01	0.01	0.02	0.02	0.01	0.00	0.00
*Difflugia protaeiformis “amphoralis”*	0.13	0.03	0.00	0.09	0.09	0.01	0.00	0.02	0.06	0.08	0.06	0.04	0.04	0.10	0.06	0.07	0.11	0.14	0.09	0.16	0.23	0.30	0.28	0.28	0.31	0.25	0.26	0.23	0.19	0.26
*Difflugia protaeiformis “acuminata”*	0.01	0.02	0.04	0.02	0.01	0.00	0.00	0.01	0.01	0.00	0.02	0.00	0.00	0.00	0.01	0.00	0.01	0.02	0.00	0.00	0.02	0.01	0.00	0.00	0.00	0.01	0.00	0.01	0.00	0.01
*Difflugia protaeiformis “scapellum”*	0.00	0.00	0.00	0.00	0.00	0.00	0.00	0.00	0.00	0.00	0.00	0.00	0.00	0.00	0.00	0.00	0.00	0.00	0.00	0.00	0.00	0.00	0.00	0.00	0.00	0.00	0.00	0.00	0.00	0.00
*Conicocassis potigulasiformis*	0.00	0.00	0.00	0.00	0.00	0.00	0.00	0.01	0.00	0.00	0.00	0.00	0.00	0.00	0.00	0.00	0.00	0.00	0.00	0.00	0.00	0.00	0.00	0.00	0.00	0.00	0.00	0.00	0.00	0.00

Species diversity was calculated using the Shannon Diversity Index (SDI) to explore changes in lake health over time (Shannon, 1948). The SDI is calculated using the formula:

\begin{eqnarray*}& & \mathrm{SDI}.=-\sum _{i=1}^{S} \left( \frac{{X}_{i}}{{N}_{i}} \right) \times \ln \nolimits \left( \frac{{X}_{i}}{{N}_{i}} \right) \end{eqnarray*}

where *X*_*i*_ is each taxon abundance in a sample, *N*_*i*_ is a sample’s total abundance, and S is the species richness of a sample. Samples were considered stable if the SDI was between 2.5 and 3.5, in transition if SDI was between 1.5 and 2.5, and stressed if SDI was below 1.5 (Magurran, 1988). Ratios between centropyxid and difflugiid species were calculated after the approach of Neville, McCarthy & MacKinnon (2010) (Table 1; Fig. S3). Stressed assemblages were defined as those with higher proportions of centropyxids to difflugiids, as centropyxid species have been found to be much more resistant to contaminated conditions as opposed to difflugiids, which have a low tolerance to contaminated conditions (Neville, McCarthy & MacKinnon, 2010; Nasser et al., 2016).

**Figure 4:**
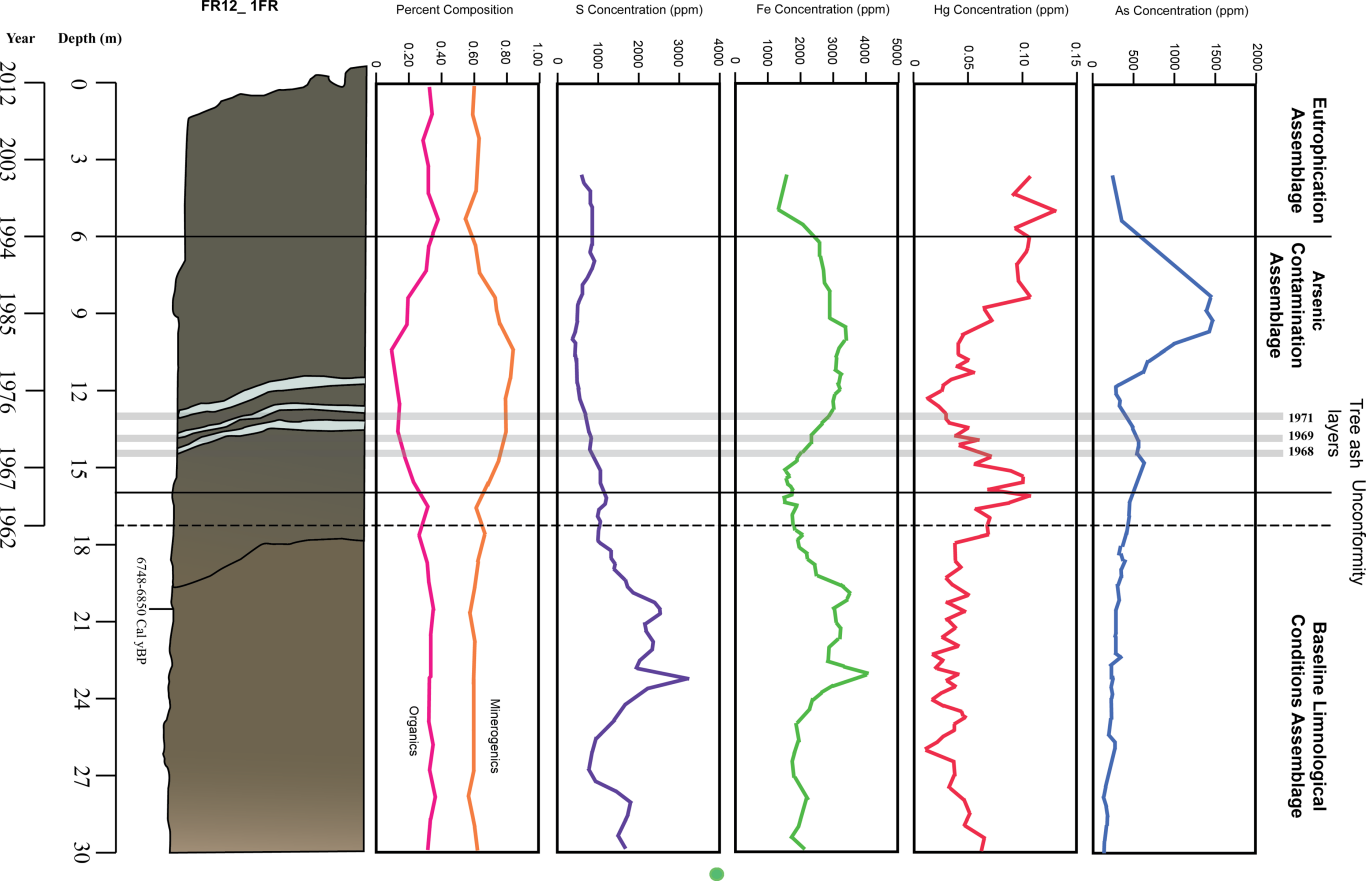
Stratigraphy and temporal variability of measured variables in Frame Lake. Stratigraphic sketch; line graphs of arsenic, mercury, iron, and sulfur concentrations; and percent composition of minerogenics and organics. Age depth was determined using ^210^Pb dating for the top 17 cm of the core and radiocarbon dates.

Geochemical data were screened prior to statistical analyses using the approach of Nasser et al. (2016) and Reimann et al. (2008). Variables that had missing values, or that were above or below the detection limit in more than 25% of their values were removed. All geochemical data was first converted to ppm and then normalized against aluminum, as it is a generally stratigraphically immobile element (Forghani et al., 2009). The LOI data were partitioned and reported as percentage water, organics, carbonates and minerogenics. Data reduction of the geochemical data was carried out using PRIMER (version 6) software by calculating Euclidean distance matrices for each element and running a 2Stage matrix (after Clarke & Gorley, 2005; Xu & Xu, 2016). Elements that were highly correlated and had no clear impact on arcellinidan assemblages were removed (after Fishbein & Patterson, 1993). Remaining elements were analyzed by using a RELATE function to run Spearman correlations, used for the typically skewed ecological datasets (i.e., datasets exhibiting a non-normal distribution), between elements and a Bray-Curtis similarity matrix of square root-transformed species counts (after Clarke & Gorley, 2005). Five variables (minerogenics, organics, arsenic, iron and mercury) that were found to be significantly correlated with species assemblages were used in the next stage of analyses (Table S2). Arsenic, mercury and iron were also compared on the basis of their ratio to sulfur and manganese to determine whether there was evidence of mobilization of elements resulting from redox reactions (Fig. 4). Further Spearman correlations were run between arsenic and elements it is often associated with; iron, manganese, and sulfur (Couture et al., 2013; Takamatsu, Kawashima & Koyama, 1985; Belzile & Tessier, 1990).

**Figure 5:**
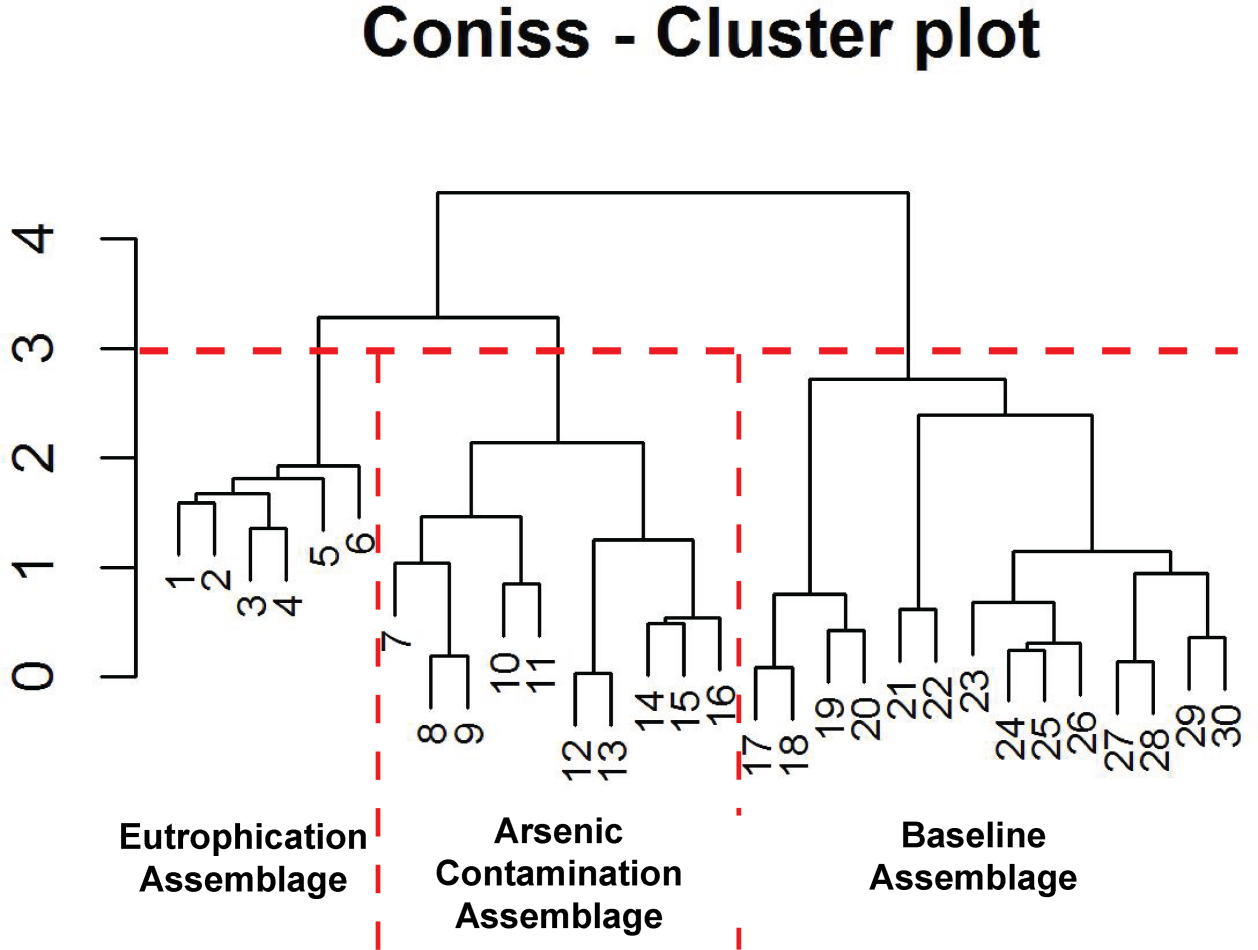
Arcellinida assemblages based on CONISS. “Broken stick” CONISS cluster plot showing three assemblages at 30% similarity.

**Figure 6:**
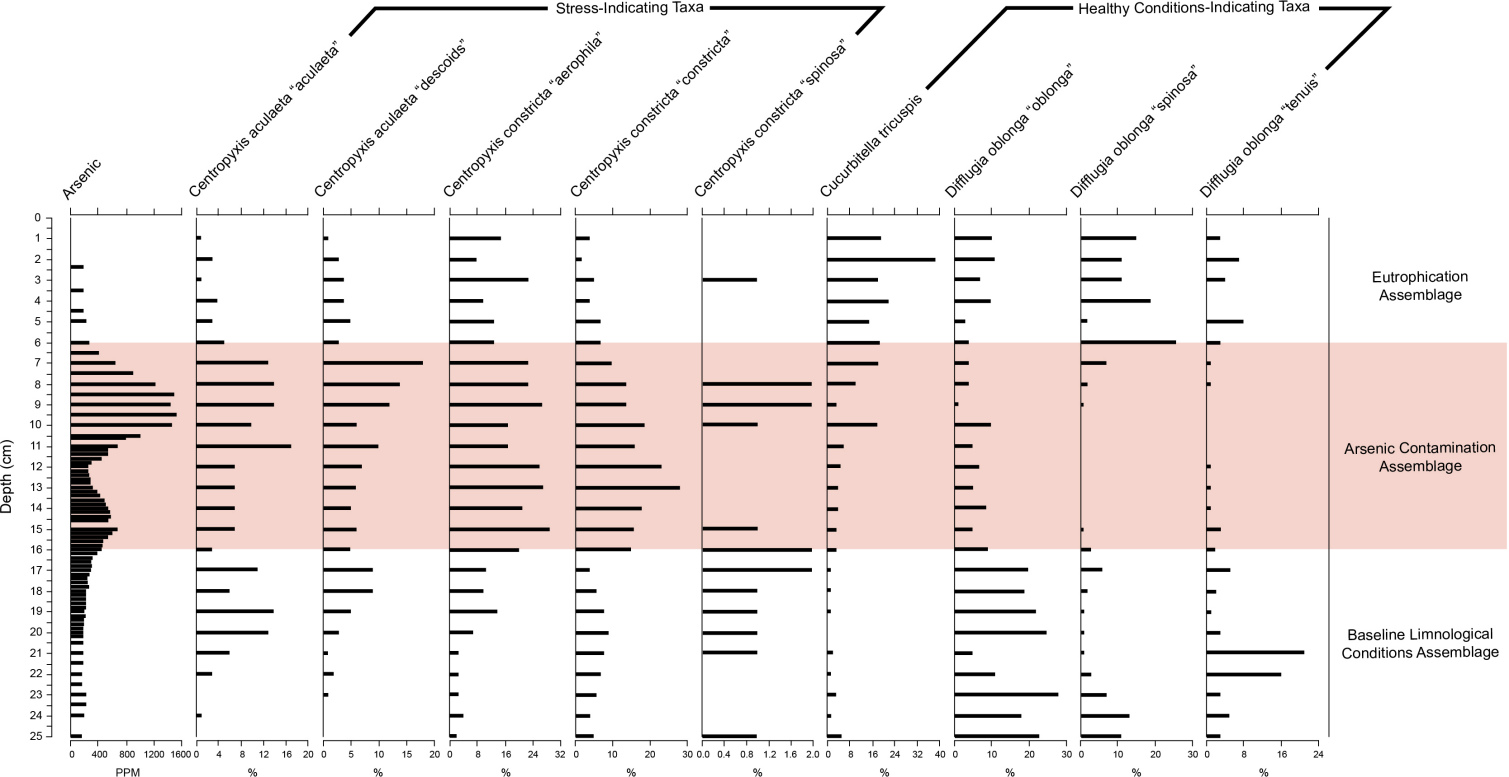
Response of key health and contamination-indicating taxa of Arcellinida to change in arsenic concentration over time. Stratigraphic profile showing arsenic concentrations over time as well as key health and contamination-indicating taxa of Arcellinida. Assemblages identified according to the CONISS and ANOSIM analysis results.

The make-up of the species assemblages was determined using CONISS cluster analysis—broken stick model in C2 (Grimm, 1987) (Fig. 5). Relationships between assemblages, core depth and arsenic were explored by creating a stratigraphic profile in C2 (Juggins, 2012) (Fig. 6). Non-metric multidimensional scaling (NMDS) function in RStudio (R Core Team, 2014) was employed to confirm the results of CONISS and assess the similarity between identified assemblages in multidimensional space (Kruskal, 1964). The one-way ANOSIM function in PRIMER was used to look for significant differences between assemblages (Clarke & Gorley, 2005). The five selected environmental variables were visually represented using line graphs created in Excel (Fig. 4).

## Results

### Chronology and sedimentology

High-resolution chronological control indicated that all AMS ^14^C dates in the upper 15 cm were of material of dateable age but were highly jumbled and most likely of an allochthonous origin (e.g., old carbon bearing sediments removed from elsewhere and dumped into the lake; old carbon bearing sediments washed into the lake from the lake catchment). Dates are presented in years before present (^14^C age BP) in Table 2 (sample at 3 cm missing). The radiocarbon date at 19.5 cm–20.5 cm was accurate as it correlated well with the known stratigraphy from lower in the core. A ^210^Pb profile from a nearby core in the same lake basin indicate that the upper 17 cm of the core dated from ∼1962 to the core collection date in 2012, and below an unconformity the underlying interval from 18 cm to 30 cm was determined to have been deposited during the early Holocene >7,000 years BP (Figs. 3 and 4; Table 3).

**Table 2:** Radiocarbon dates for top 20.5 cm of core.

Interval (cm)	C14 age (BP)	±	F14C	±
0.5	405	20	0.9508	0.0024
1	591	19	0.9290	0.0022
1.5	513	19	0.9382	0.0023
2	536	20	0.9354	0.0023
2.5	657	20	0.9215	0.0022
3	–	–	–	–
3.5	853	20	0.8993	0.0022
4	1,044	21	0.8781	0.0022
4.5	1,005	20	0.8824	0.0022
5	661	19	0.9211	0.0021
5.5	349	18	0.9574	0.0022
6	614	19	0.9264	0.0022
6.5	788	21	0.9065	0.0023
7	373	19	0.9546	0.0022
7.5	1,070	18	0.8753	0.0020
8	865	21	0.8979	0.0024
8.5	519	19	0.9375	0.0022
9	635	19	0.9240	0.0022
9.5	955	21	0.8879	0.0023
10	749	19	0.9110	0.0021
10.5	945	19	0.8891	0.0021
11	1,012	23	0.8816	0.0025
11.5	872	22	0.8971	0.0025
12	1,056	19	0.8768	0.0021
12.5	846	20	0.9001	0.0023
13	736	25	0.9125	0.0028
13.5	866	19	0.8978	0.0021
14	1,123	64	0.8695	0.0069
14.5	1,049	45	0.8776	0.0049
15	1,233	20	0.8578	0.0021
19.5–20.5	7,230	128		

**Table 3:** Ages of core based on ^210^Pb age model extrapolated to this core from core results taken from a fourth core extracted from the same basin in 2014 and known dates (1968, 1969 and 1971) from wood ash stratigraphic units in the south basin core under examination (former Yellowknife Mayor, D Lovell, pers. comm., 2015).

Depth	Sed. rate-Pb201 (cm/yr)	Age-Pb210 (yr)	Sed. rate ash (cm/year)
0	1.5	2012.00	0.34
1	0.48	2011.33	0.34
2	0.71	2009.23	0.34
2.5	0.99	2008.53	0.34
3	1.13	2008.02	0.34
3.5	1.18	2007.58	0.34
4	0.75	2007.16	0.34
4.5	0.52	2006.48	0.34
5	0.20	2005.52	0.34
6	0.43	2000.47	0.34
7	0.43	1998.12	0.34
8	0.45	1995.81	0.34
9	0.60	1993.60	0.34
10	0.45	1991.94	0.34
11	0.36	1989.70	0.34
12	0.24	1986.96	0.34
13	0.24	1982.74	0.34
14	0.24	1978.64	0.34
15	0.17	1974.47	0.34
16	0.17	1968.58	0.34
17	0.17	1962.69	0.34
18	0.17	1956.81	0.34
19	0.17	1950.92	0.34
20	0.72		0.34

The 1FR core was characterized by darker sediments (5Y 2.5/1) in the top 44 cm of the core, with an ash layer occurring between 14 and 15 cm (Figs. 3 and 4). Further refining of the age model was possible due to the presence of three prominent stratigraphic markers comprised of wood ash with known depositional dates. These ash layers were derived from the burning of discarded Christmas trees in bonfires on the lake as part of New Year’s Eve celebrations in 1968, 1969 and 1971 (Figs. 3 and 4; Table 3) (V Sterenberg, former Yellowknife mayor, D Lovell, pers. comm., 2015). An unconformity at 17 cm marked the boundary between early Holocene sedimentation (7,230 ± 128) and sediments deposited after 1962 (Table 3; Figs. 3 and 4).

### Loss on ignition

The loss on ignition (LOI) data (Table 1) showed an increase in both water and minerogenic content between the 6 and 17 cm core intervals. Organic content was correspondingly lower through this interval. Carbonate concentration remained stable throughout the entire 30 cm core interval studied. The minerogenic content were found to be the most significantly correlated environmental variable with arcellinida assemblages, with a Spearman’s Rho value of 0.317 and a *p* value of 0.002 (*n* = 30 samples) (Table S2; Fig. 4). Organics were calculated to be the second highest ranked environmental variable correlated with arcellinidan assemblages with a Spearman’s Rho value of 0.228 and a *p* value of 0.004 (Table S2; Fig. 4).

### Arcellinida

#### (a) CONISS, ANOSIM and NMDS

**Figure 7:**
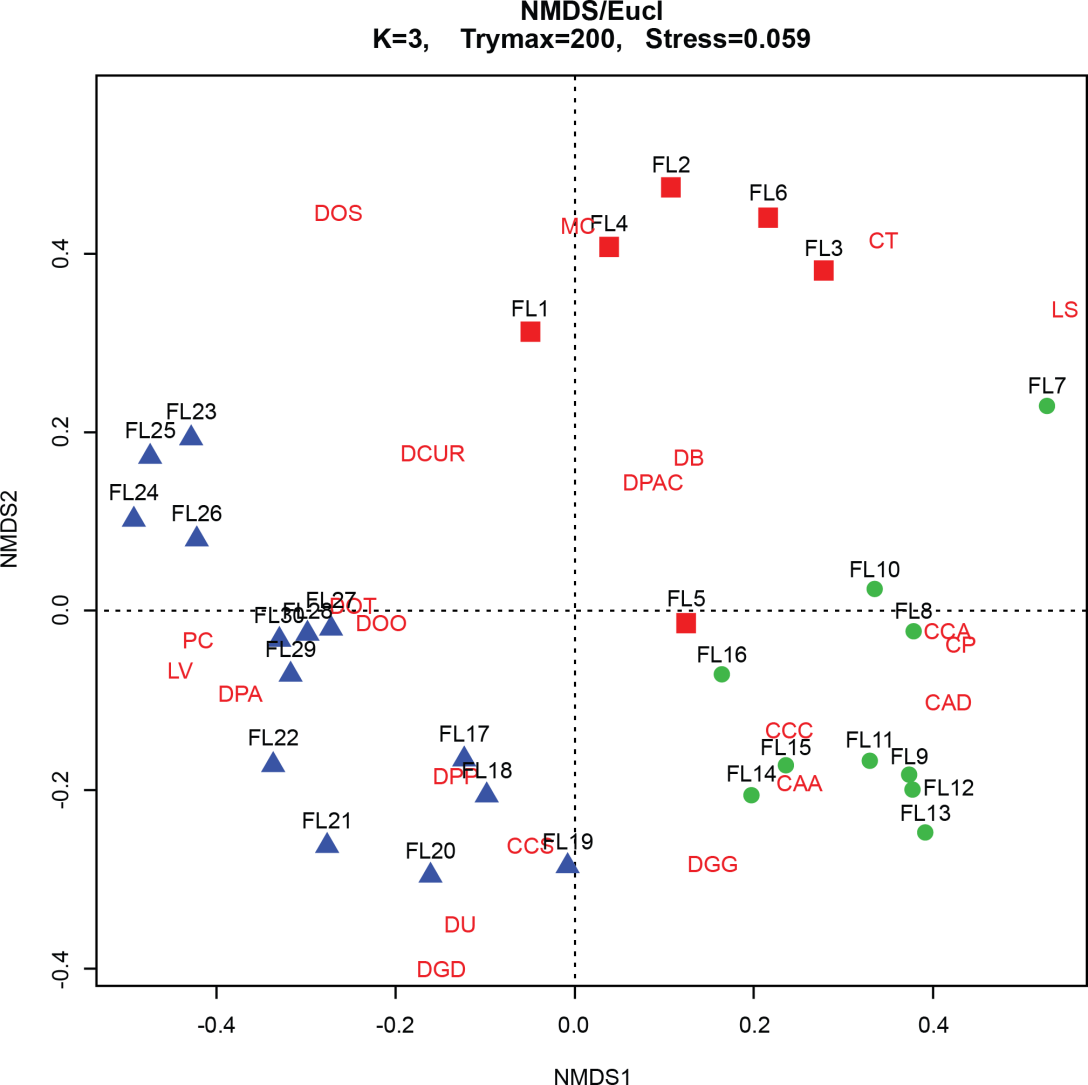
Non-Metric Multidimensional Scaling (NMDS) bi-plot. CAA, Centropyxis aculeata “aculeata”; CAD, Centropyxis aculeata “discoides”; CCA, Centropyxis constricta “aerophila”; CCC, Centropyxis constricta “constricta”; CCS, Centropyxis constricta “spinosa”; CP, Centropyxis pontigulasiformis; CT, Cucurbitella tricuspis; MC, Mediolus corona; DOO, Difflugia oblonga “oblonga”; DOS, Difflugia oblonga “spinosa”; DOT, Difflugia oblonga “tenuis”; DGG, Difflugia glans “glans”; DGD, Difflugia glans “distenda”; DU, Difflugia urens; DPP, Difflugia protaeiformis “protaeiformis”; DPA, Difflugia protaeiformis “amphoralis”; DPAC, Difflugia protaeiformis “acuminata”; DCUR, Difflugia curvicaulis; LS, Lesquereusia spiralis; LV, Lagenodifflugia vas; PC, Pontigulasia compressa.

The CONISS—cluster plot (Fig. 5) displayed three distinct assemblages at the 30% similarity mark. Assemblage 1 spanned a depth of 17–30 cm, assemblage two spanned a depth of 7–16 cm and assemblage three spanned a depth of 1–6 cm. Three distinct assemblages were identified by the NMDS bi-plot and matched CONISS results with the exception of sample FL5, with characteristics of both Assemblages 2 and 3, found at the stratigraphic boundary of these two assemblages. The results of the one-way ANOSIM test and comparison to the CONISS-defined species assemblages indicated that the assemblages were significantly different with global *R* statistic of 0.832 and a *p* value of 0.001 (*n* = 30 samples, *n* = 32 species). These results were further corroborated by the NMDS bi-plot, which revealed three groups of samples clustering closely to form the three identified arcellinidan assemblages (Fig. 7).

#### (b) Stratigraphic profile

The stratigraphic profile (Fig. 6) showed that stress-indicating taxa (centropyxids) were more abundant when arsenic levels were more elevated, while species like *Cucurbitella tricuspis* (Carter, 1856) thrived in sediments with low arsenic concentrations. Other taxa such as *Difflugia oblonga* (Ehrenberg, 1832) strain “oblonga”, *Difflugia oblonga* (Ehrenberg, 1832) strain “spinosa” were more abundant in Assemblages 1 and 3, also corresponding to the decrease in arsenic concentrations.

#### (c) Shannon diversity and difflugiid/centropyxid ratio

SDI calculations (Table 1) revealed that almost all samples were “in-transition” with the exception of samples 5, 16–18 and 28 that were in the stable range (Shannon, 1948; Patterson et al., 2013). The relative abundances of difflugiids (vs. centropyxids) fell below 0.55 between samples 7–16, coinciding with Assemblage 2 (Fig. S3). The difflugiid/centropyxid ratio remained above 0.55 for Assemblages 1 and 3, and showed an increase over time in Assemblage 3 (Table 1; Fig. S3) (Neville, McCarthy & MacKinnon, 2010).

### Geochemical variables

Arcellinida abundances were significantly correlated with arsenic (Table S2) with the third-ranked Rho value of 0.196 and *p* value of 0.034. Arsenic levels showed an increase over time, peaking at 15 and 8 cm, but showed a drop at 12 and 5 cm (Figs. 4 and 6). Arcellinida also showed a significant correlation with iron with an *R*-value of 0.192 and *p* value of 0.019 (Table S2). Iron levels peak at 24 cm but begin to show a marked decrease up-core of 20 cm (Fig. 4). Mercury was also found to be significantly correlated with arcellinidan abundances with an *R*-value of 0.168 and a *p* value of 0.046 (Table S2). Mercury values peak at 16 cm, then drop at 12 cm, but peak once again at 5 cm (Fig. 4). A comparison of mercury and arsenic distribution through the core to that of manganese, iron and sulfur indicated the second peaks of mercury and arsenic were possibly due to the mobilization of the elements (Fig. 4).

The correlation between arsenic and iron was significant (*R* = 0.336, *p* = 0.001, *n* = 140), as well as the correlation between arsenic and manganese (*R* = 0.093, *p* = 0.002, *n* = 140). The correlation between arsenic and sulfur was not found to be significant at *R* =  − 0.059, *p* = 0.954, *n* = 140.

### Aerial photos

Aerial photography capturing eight decades of urbanization around Frame Lake indicated that while the area around the lake was relatively undisturbed in 1937, it had major development by 1964 (Fig. 2).

### Assemblages

The results of the ANOSIM test, Spearman correlations, NMDS and CONISS cluster analyses demonstrate that Arcellinida sample assemblages could be statistically separated into three distinct groups, which correlated closely with their response to environmental conditions (Fig. 5; Table S2). Assemblage 1 (Baseline Limnological Conditions Assemblage [BLCA]) characterized samples deposited below the unconformity during the early Holocene. Assemblage 2 (Arsenic Contamination Assemblage [ACA]) was associated with mine waste influence and Assemblage 3 (Eutrophication Assemblage [EA]) developed during the more recent decades when deposition of mine contaminants ceased but level of nutrient input to the lake increased.

### Baseline Limnological Conditions Assemblage—BLCA (*n* = 14)

The BLCA ranges from samples 17 to 30 and corresponds to ancient sediments deposited in the lake basin in the early Holocene, >7,000 years BP (Figs. 3 and 4). The SDI indicated that samples in this assemblage were all in transition, with the exception of samples 17, 18 and 28 that were in the stable range (Table 1) (Shannon, 1948; Patterson et al., 2013). Samples 17 and 18 mark the location of the unconformity between early Holocene and 20th century horizons in the lake, and sample 28 may mark an ecological transitional zone representing early hydroecological conditions as the lake transitioned from a basin within the very large Proto-Great Slave Lake into a small isolated lake. The difflugiid/centropyxid ratio revealed a strong dominance by difflugiids with a mean of 0.78 (Table 1; Fig. S3) (Neville, McCarthy & MacKinnon, 2010). Samples comprising the BLCA clustered together at both the 30% and 40% similarity marks, as seen in the CONISS plot and confirmed by the NMDS bi-plot (Figs. 5 and 7).

The BLCA is characterized by stable levels of minerogenics and organics, very similar to those found in the EA (Fig. 4, Table 1). Arsenic concentrations corresponding to the BLCA remain relatively stable, ranging from 289.8 to 121.4 ppm (Figs. 4 and 6). Mercury concentrations in the BLCA show a steady trend of short peaks and drops, but remain relatively constant throughout the interval (Fig. 4). Iron concentrations show a steady increase in the BLCA, peaking at 23 cm, but sharply dropping at 26 cm (Fig. 4). The faunal diversity of the BLCA is dominated by *Difflugia protaeformis* (Ehrenberg, 1830) strain “amphoralis” (22%), *D. oblonga* “oblonga” (19%) and *D. oblonga* “spinosa” (8%) (Table 1).

### Arsenic Contamination Assemblage—ACA (*n* = 10)

The ACA spans samples 7–16 and corresponds to deposition between ∼AD 1962 and 1994 (Figs. 3 and 4; Table 3). The SDI indicates that these samples are in transition, with the exception of sample 16 that was in the stable range (2.6), marking an ecological transitional zone between the ACA and BLCA (Table 1) (Shannon, 1948; Patterson et al., 2013). The difflugiid/centropyxid ratio showed a significant drop in difflugiid abundances, with a mean value of 0.33 (Table 1; Fig. S3) (Neville, McCarthy & MacKinnon, 2010). The ACA clustered together in the CONISS plot (Fig. 5), indicating samples 7–16 are similar at both the 30% and 40% levels. Samples hosting this assemblage were also shown to exhibit tight grouping when examining the NMDS bi-plot. Interestingly, while sample FL7 appear to be part of this assemblage based on the CONISS results, the NMDS bi-plot revealed a notable break between the sample and the assemblage (Fig. 7). This could be attributed to the fact that the assemblage composition of FL7 is notably different than that of ACA.

The ACA is characterized by rising levels of minerogenics that peak at 11 cm and correspondingly low levels of organics that hit their lowest concentrations at 10 cm (Fig. 4, Table 1). Arsenic levels in the ACA show a double peak at 8.5 and 15 cm as well as a drop at 12 cm (Figs. 4 and 6). Mercury levels in the ACA show a double peak at each end of the assemblage interval and also have a marked drop at 12 cm (Fig. 4). Iron levels remain relatively constant throughout the ACA interval (Fig. 4). The faunal diversity of the ACA is characterized primarily by *Centropyxis constricta* (Ehrenberg, 1843) strain “aerophila” (23%), *Centropyxis constricta* (Ehrenberg, 1843) strain “constricta” *Centropyxis aculeata* (Ehrenberg, 1832), strain “aculeata” and *Centropyxis aculeata* (Ehrenberg, 1832), and strain “discoides” (Table 1).

### Eutrophication Assemblage—EA (*n* = 6)

The EA is comprised of samples that range from 1 to 6 cm and corresponds to deposition in the lake from 1994 to 2012 (Figs. 3 and 4; Table 3). The SDI indicated that EA samples are in transition, with the exception of sample 5 that was in the stable range (Table 1) (Shannon, 1948; Patterson et al., 2013). The difflugiid/centropyxid ratio showed a dominance of difflugiid species with all values over 0.55 (Table 1; Fig. S3) (Neville, McCarthy & MacKinnon, 2010). The CONISS plot demonstrated that samples in the EA showed strong similarity as they clustered together at the 30% similarity range (Fig. 5). This similarity is also salient in the results of NMDS with the exception of sample FL5 plotting close to the ACA (Fig. 7). This is not surprising as the faunal composition of the sample is generally similar to that of the EA while also hosting a notable number of centropyxid species that are more characteristic of the ACA.

The EA corresponds to stable levels of organics, minerogenics and iron (Fig. 4, Table 1). Arsenic levels in the EA show a continuous decrease while mercury levels show a peak closer to the ACA and then drop further up core (Figs. 4 and 6). The faunal diversity of the EA is comprised mostly of *C. tricuspis, C. constricta* “aerophila”, *D.* oblonga “spinosa”, and *D. oblonga* “oblonga” (Table 1).

## Discussion

The results of the ANOSIM, CONISS-Cluster models and NMDS clearly indicate a temporal differentiation between the identified Arcellinida assemblages (Figs. 5 and 7). The assemblages correlate to distinct hydroecological states and indicate a particularly distinct relationship between assemblages and anthropogenic impact (Table 3, Fig. 4). Arcellinida have previously been used to document land use changes by Patterson & Kumar (2002); Patterson et al. (2002) and geochemical loading in lakes surrounding the Yellowknife area by Nasser et al. (2016). By investigating the species and strains that are dominant in each assemblage, as well as the dominant significant environmental variables, it is possible to determine the impact that legacy mining activities had on geochemical loading of the lake and the role of urbanization on the hydroecologic development of Frame Lake.

The oldest assemblage, the BLCA, is not part of the Yellowknife urbanization narrative, as these sediments were deposited in an ancient lake environment, dating back more than 7,000 years (Figs. 3 and 4). These sediments are characterized by higher levels of iron (Fig. 4) and high abundances of *D. protaeformis* “amphoralis”, *D. oblonga* “oblonga”, as well as *D. oblonga* “spinosa” (Table 1). *Difflugia protaeformis* “amphoralis” is most commonly found at present in muddy sediments, characterized by high numbers of pennate diatoms thought to be their preferred food source (Reinhardt et al., 1998; Patterson & Kumar, 2000a; Patterson & Kumar, 2000b). As observed in this research, *D. protaeformis* “amphoralis” was also seen in pre-disturbance sediments by Kihlman & Kaupila (2012). *D. oblonga* “oblonga” are very common globally, ranging from the Arctic to the tropics, provided there are enough organics in the substrate. *D. oblonga* “oblonga” species are commonly found in mesotrophic, nutrient-rich, healthy lake environments (Reinhardt et al., 1998; Patterson & Kumar, 2000a; Patterson & Kumar, 2000b; Roe & Patterson, 2014).

Healthy lake conditions in the BLCA are further indicated by the presence of *D. oblonga* “spinosa”. *D. oblonga* “spinosa” are commonly found in environments characterized by vegetated clay or silt-clay sediments (Reinhardt et al., 1998). It has also been suggested that *D. oblonga* “spinosa” prefer higher nutrient levels and higher concentrations of dissolved oxygen (Roe, Patterson & Swindles, 2010; Kihlman & Kaupila, 2012). The SDI values in the BLCA were high ($\overline{x}=2.41$), indicating a good species distribution and a high abundance of species (Patterson & Kumar, 2002; Patterson et al., 2002). A high SDI value, coupled with a high proportion of difflugiids to centropyxids ($\overline{x}=0.78$) (Table 1) further support that the BLCA characterizes a healthy lake environment (Patterson & Kumar, 2002; Neville, McCarthy & MacKinnon, 2010).

Directly above the BLCA assemblage is an unconformity at 17 cm, separating the >7,000-year-old sediments from the modern deposition at this site, which began in 1962 (Figs. 3 and 4; Table 3). The reason for the hiatus/erosion of most of the Holocene record in the lake is hypothesized to be due to the gradual recession of Frame Lake that gradually isolated the basin from nearby Yellowknife Bay. When connected to Yellowknife Bay, Frame Lake received sediment from the nearby Yellowknife and Cameron rivers. However, as Frame Lake became progressively more shallow and isolated, the sedimentation levels would have dropped (Wolfe & Morse, 2015). It seems that the initiation of urbanization around Frame Lake “turned on” the depositional system within the lake, allowing it to transition from a non-depositional environment, to a depositional catchment characterized by high sedimentation rate ($\overline{x}=0.52$ cm/year). The post 1962 sediments are characterized by the ACA. This assemblage is characterized by a drop in SDI values ($\overline{x}=2.33$) and a low ratio of difflugiid to centropyxids ($\overline{x}=0.33$) (Table 1; Fig. S3), indicating environmental conditions unfavourable to Arcellinida (Neville, McCarthy & MacKinnon, 2010).

The ACA is dominated by centropyxid species. Centropyxids have been demonstrated to be generalists, capable of living in stressed environments (Patterson, Barker & Burbidge, 1996; Reinhardt et al., 1998; Patterson & Kumar, 2000a; Patterson & Kumar, 2000b). Frame Lake began to act as a catchment to sediments in 1962 (Table 3; Figs. 3 and 4). By this time, the surrounding landscape would have received arsenic via aerial deposition from the Giant Mine (Government of Canada, 2013a). Disturbance of the landscape in the Frame Lake catchment as Yellowknife began to expand into the area may have resulted in residual arsenic from roaster stack emissions that were sequestered on the landscape washing into the lake (e.g., Fawcett et al., 2015; Van Den Berghe, Jamieson & Palmer, 2017). Additional sources of arsenic may have been derived from dumping of arsenic contaminated sediments from elsewhere in the area, or deposition of sediments from the catchment with naturally elevated arsenic levels (Figs. 4 and 6). Whatever the origin there is an arsenic peak stratigraphically positioned in sediments deposited centered around 1967 in the core (Figs. 4 and 6). A second arsenic peak centered around 1985 is most likely attributable to arsenic mobilization towards more oxic conditions above the redox line (Fig. 4) (Boyle, 2002; Neff, 2002; Van Den Berghe, Jamieson & Palmer, 2017). Arsenic concentrations as high as 1,505 ppm, were measured, far exceeding the Canadian Council of the Ministers of the Environment (CCME) probable effect levels for freshwater sediment of 17 ppm, as well as the interim sediment quality guideline of 5.9 ppm (CCME, 1999). Arsenic re-mobilization in lake sediments from Yellowknife lakes impacted by legacy gold mining has also been documented by Andrade et al. (2010) and Schuh et al. (in press). The high arsenic concentrations are associated with a peak in centropyxid abundances, although the respective peaks do not align (Figs. 4 and 6, Fig. S3; Table 1). The resilience of centropyxids to metal(loid) contaminants such as arsenic has been demonstrated by Patterson, Barker & Burbidge (1996) and most recently by Nasser et al. (2016). Unlike arsenic, which is responsive to changes in redox conditions and therefore mobile in core records, the stratigraphically immobile bioindicator the centropyxid peak indicates the actual temporal position of maximum contaminant levels. This lack of vertical mobility of Arcellinida through sediment horizons illustrates their importance as bio-indicators of metal(loid) contamination. Mercury, although plentiful in lake sediments and also known to be affiliated with the gold extraction process (DIAND, 2007), was not included in our narrative of the events surrounding the sedimentation of Frame Lake due to the potential for inaccurate readings from the analytic mercury memory effect and mass spectral interferences using ICP-MS determination for this element (Li et al., 2006). The increased concentrations of iron throughout the ACA can be attributed to its high binding affinity with arsenic (Takamatsu, Kawashima & Koyama, 1985), as reflected in the high correlation between the two.

The ACA is also characterized by higher levels of minerogenics (Fig. 4, Table 1), that correspond to the relatively high sedimentation rate in Frame Lake that developed through this interval (∼1962–1994). Multiple anthropogenic events around Frame Lake correlate to the spike in sedimentation rate. The population of Yellowknife increased from about 1,000 people in the 1950s to over 3,500 by the early 1960s. This population increase resulted in the development of “New Town”, which grew up adjacent to Frame Lake. In the 1970s, the lake was used as a snow dump, leading to a build-up of sediments in the lake (former Yellowknife mayor, D Lovell, pers. comm., 2015). The dumping of snow may also provide an explanation for the presence of mixed radiocarbon dates from sediments deposited during this interval, which may have been influenced by an influx of ‘old carbon’ derived from terrestrial material swept up by snow plows moving through the community (Table 2). The mixed radiocarbon dates could also reflect other anthropogenic activities such as construction wherein material was dug up elsewhere and deposited into the lake. Further sedimentation from urbanization events and construction around the lake, such as the development of a causeway, continued to contaminate the system (Fig. 2) (V Sterenberg, former Yellowknife mayor, G Van Tighem, pers. comm., 2015), contributing to the stressed conditions reflected in the ACA.

Deposited on top of the ACA, the EA represents a more recent shift in hydroecologic conditions in the lake (post-1990 deposition). The EA is primarily characterized by increased populations of *C. tricuspis* (Table 1, Fig. 6)*.* The presence of *C. tricuspis* has been interpreted to indicate eutrophic water conditions and a system undergoing slow remediation (Collins et al., 1990; Patterson et al., 2013). The increase in *C. tricuspis* populations, which make up a progressively higher percentage of the arcellinida population up-core, can be attributed to an ever increasing nutrification of Frame Lake. Nutrients enter the lake from many sources, including input from nearby subdivisions, run-off from adjacent parking lots, as well as storm water outflow (V Sterenberg, former Yellowknife mayor, G Van Tighem, pers. comm., 2015). The urbanization of the area around Frame Lake also changed the inflow/outflow of the lake, creating a more closed endorheic system, allowing nutrients to accumulate. In particular, the construction of a major road between 1948 (Fig. S4) and 1964 (Fig. 2), which later became a causeway in 1975, through a slowdown in outflow resulted in a major restriction of lake water circulation, exacerbating the effects of dumping and development. The results of nutrification are mirrored in the explosion in *C. tricuspis* populations in recent years.

Eutrophication events have previously been documented by Brunskill, Grahaman & Rudd (1980) to be exacerbated by arsenic contamination. During peak arsenic contamination, the degradation of organics during winter months would have been restrained due to the toxic influence of arsenic and its disruption of organic matter, possibly leading to a relatively steady buildup of organics over time (Brunskill, Grahaman & Rudd, 1980). As arsenic became less abundant in the system, rejuvenated microbial degradation of residual organics could have impacted the de-oxygenation of the lake (Brunskill, Grahaman & Rudd, 1980). The eutrophication of lakes has been well documented to result from excess sedimentation and nutrient input (Carpenter, Ludwig & Brock, 1999; Qin, 2009; Le et al., 2010). As the area around Frame Lake became increasingly urban, the accumulation of nutrients and sediments in the lake began to render the lake more eutrophic. The process was exacerbated by the low in/outflow of water to the lake (Dirszowsky et al., 2013).

As Frame Lake became progressively more dysoxic in winter, fish populations were no longer able to survive, especially during the ice-cover months. Fish-kills as the result of eutrophication have been seen in multiple other studies (see Tammi et al., 1999; Kangur et al., 2013). Fishes, such as Lake Whitefish, Northern Pike, and suckers that were known to previously occur in Frame Lake (R Freeman, Chief Fred Sangris, pers. comm., 2015; Yellowknives Dene First Nations, 2016), consume various invertebrates including leeches (Scott & Crossman, 1973). With predation by fishes reduced and eventually eliminated leeches would have experienced a dramatic increase in abundance. Leeches have been previously documented to thrive in systems that have experienced fish-kills (Wen, 1992). In only a few years this increase in leech populations quickly drove swimmers away from McNiven Beach, a once popular recreational area along Frame Lake’s shores (CBC, 2015; EDGE North, 2015). It is worth noting that leeches in Frame Lake are very large and can exceed 15 cm in length (P Cott, pers. obs., 2016).

The progressive nutrification of Frame Lake has also recently been documented by Dirszowsky & Wilson (2016). Consistent with our findings, Dirszowsky & Wilson (2016) found that Frame Lake was exceptionally susceptible to eutrophication due to its limited depth and turnover rates. Both our studies illustrated that the sediments contained high levels of arsenic, reflecting the legacy of gold mining in the surrounding area, as well as contamination from development around the lake, and both our sediment cores showed that arsenic concentrations have since dropped, with a concomitant increase in organics. There was a difference in the interpretation of the sediment chronology of the lake as Dirszowsky & Wilson (2016) did not have radiocarbon chronology and erroneously based their estimates of the age of the BLCA sediments on a downward extrapolation of their ^210^Pb age profile.

## Conclusions

Arcellinida assemblages were found to be an important indicator of the impact of land use change on the hydrology of Frame Lake, and indicated that the stratigraphic sequence studied could be differentiated into three distinct hydroecological units: (1) Baseline Limnological Conditions Assemblage [BLCA]) characterized samples deposited below the unconformity during the early Holocene (>7,000 years BP); (2) Arsenic Contamination Assemblage [ACA]) associated with aerial deposition from mining processes, possible illegal sediment dumping, and urbanization around Frame Lake from 1962 onward when deposition of sediments was reinitiated in the lake; and (3) Eutrophication Assemblage [EA]) developed during the more recent decades when deposition of mine contaminants ceased. Of particular significance to urban planners, the EA indicates that Frame Lake has become increasingly eutrophic as a result of changes to runoff associated with urbanization around the lake shore and disruption to circulation following construction of roadways across the lake outflow, particularly the building of the current causeway in 1975. This eutrophication eventually led to extensive macrophyte growth and winter fish-kills, ultimately creating a system where the leech population thrived. Further research around the lake should be conducted to measure nutrient input levels from connecting streams. Based on these results possible remediation/mitigation strategies being explored for Frame Lake include dredging of contaminated sediments to remove arsenic, biologically digesting contaminated sludge using aeration, the construction of a storm water management pond to reduce the input of nutrients to the lake, and opening of the causeway gates to increase water flow through the lake system. By implementing these strategies, winter fish-kills can be mitigated, helping to rehabilitate the lake and allowing it to support fish populations year-round.

##  Supplemental Information

10.7717/peerj.4850/supp-1 Figure S1 Photograph taken at McNiven Beach in 1967, illustrating the popularity the beach once had Photo by Ted Grant. NWT Archives, Northwest Territories. Department of Information fonds, accession number G-1979-023, item number 0146.

10.7717/peerj.4850/supp-2 Figure S2 Photo of RT Patterson demonstrating the extensive macrophyte cover at Frame Lake during the 2015 field season Photo by N.A. Nasser.

10.7717/peerj.4850/supp-3 Figure S3 Air photo of Frame Lake and surrounding area in 1948 (A11544-009, NAPL 2017)

10.7717/peerj.4850/supp-4 Figure S4 Difflugia/centropyxis ratio

10.7717/peerj.4850/supp-5 Table S1 Ranked Rho values for Spearman rank correlations with significance

10.7717/peerj.4850/supp-6 Table S2 Ranked Rho values for Spearman rank correlations with significance

10.7717/peerj.4850/supp-7 Data S1 Raw data

## Additional Information and Declarations

### Data Availability

The following information was supplied regarding data availability: The raw data are provided in Data S1.
