# Supplementary figures and images for: What killed Frame Lake? A precautionary tale for urban planners
Source: PeerJ. 2018 Jun 14;6:e4850. doi: 10.7717/peerj.4850 (PMC6004302; doi:10.7717/peerj.4850)

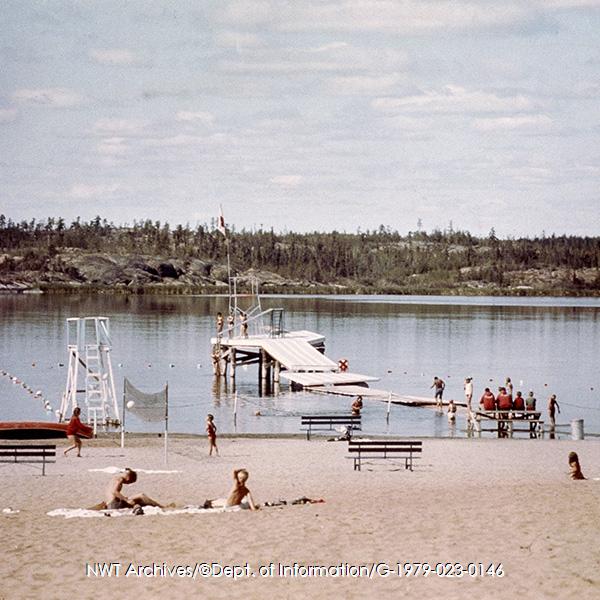

Supplement: Figure S1 — Photo by Ted Grant. NWT Archives, Northwest Territories. Department of Information fonds, accession number G-1979-023, item number 0146. [file peerj-06-4850-s001.jpg]

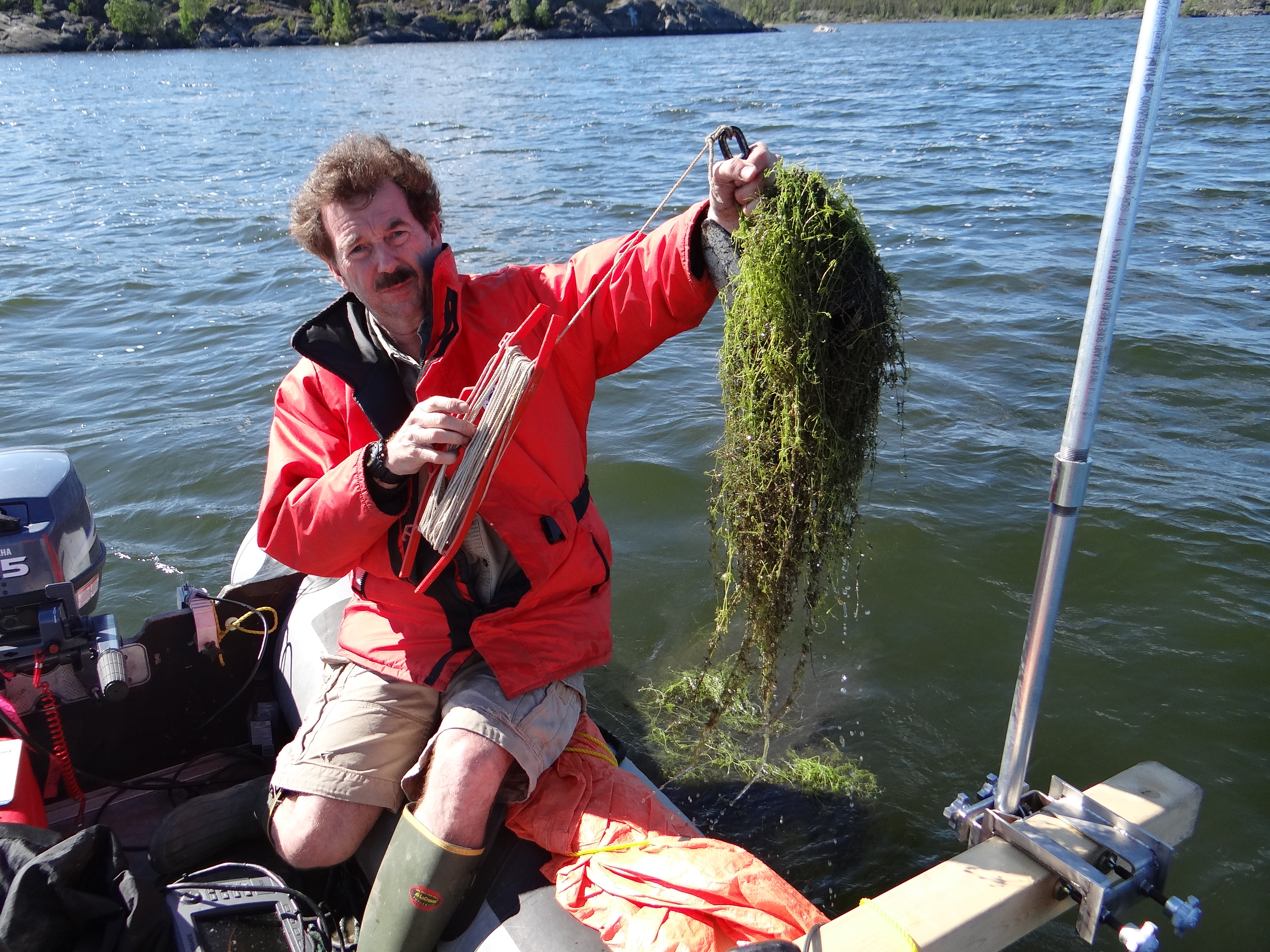

Supplement: Figure S2 — Photo by N.A. Nasser. [file peerj-06-4850-s002.jpg]

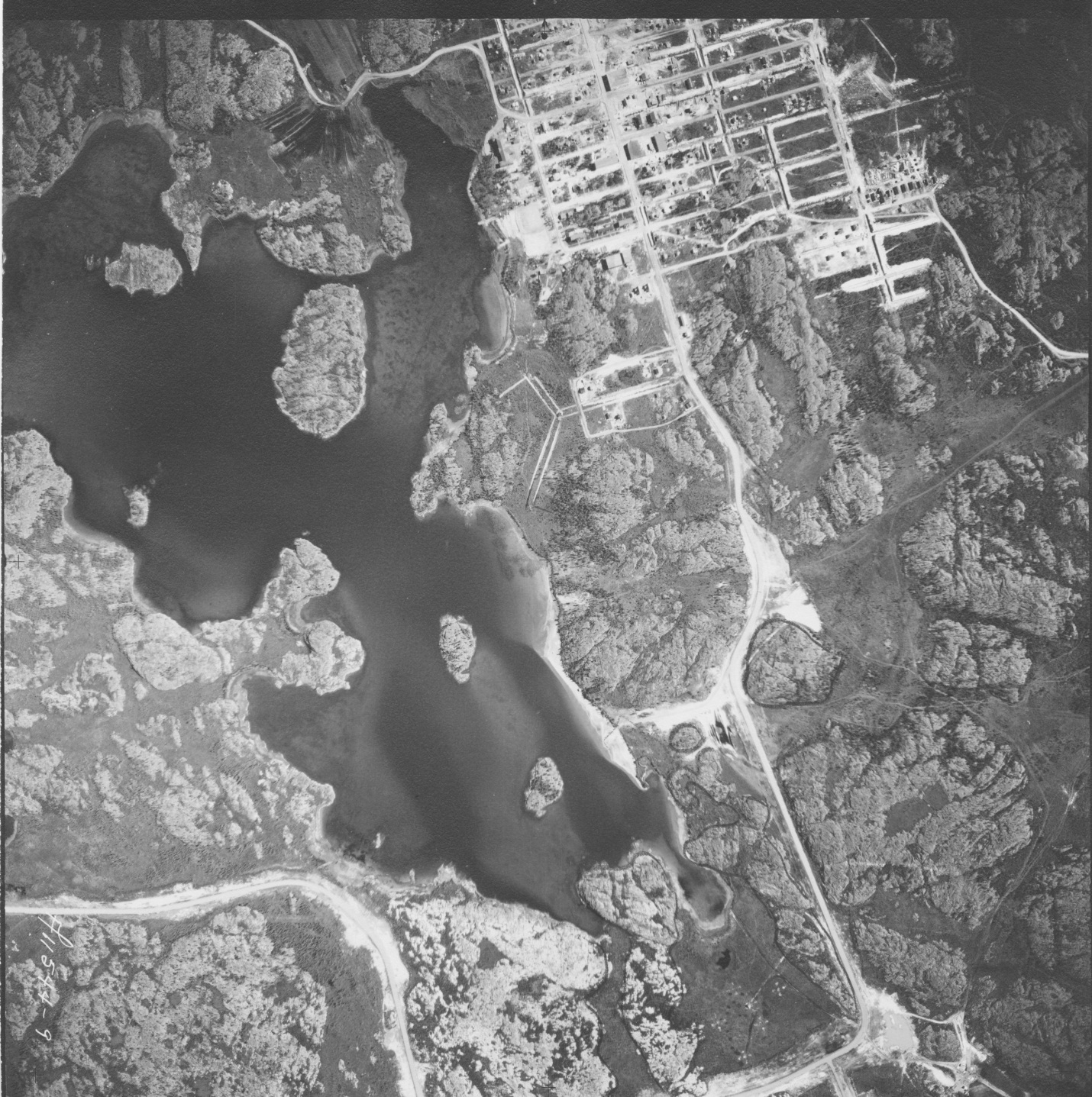

Supplement: Figure S3 [file peerj-06-4850-s003.jpg]

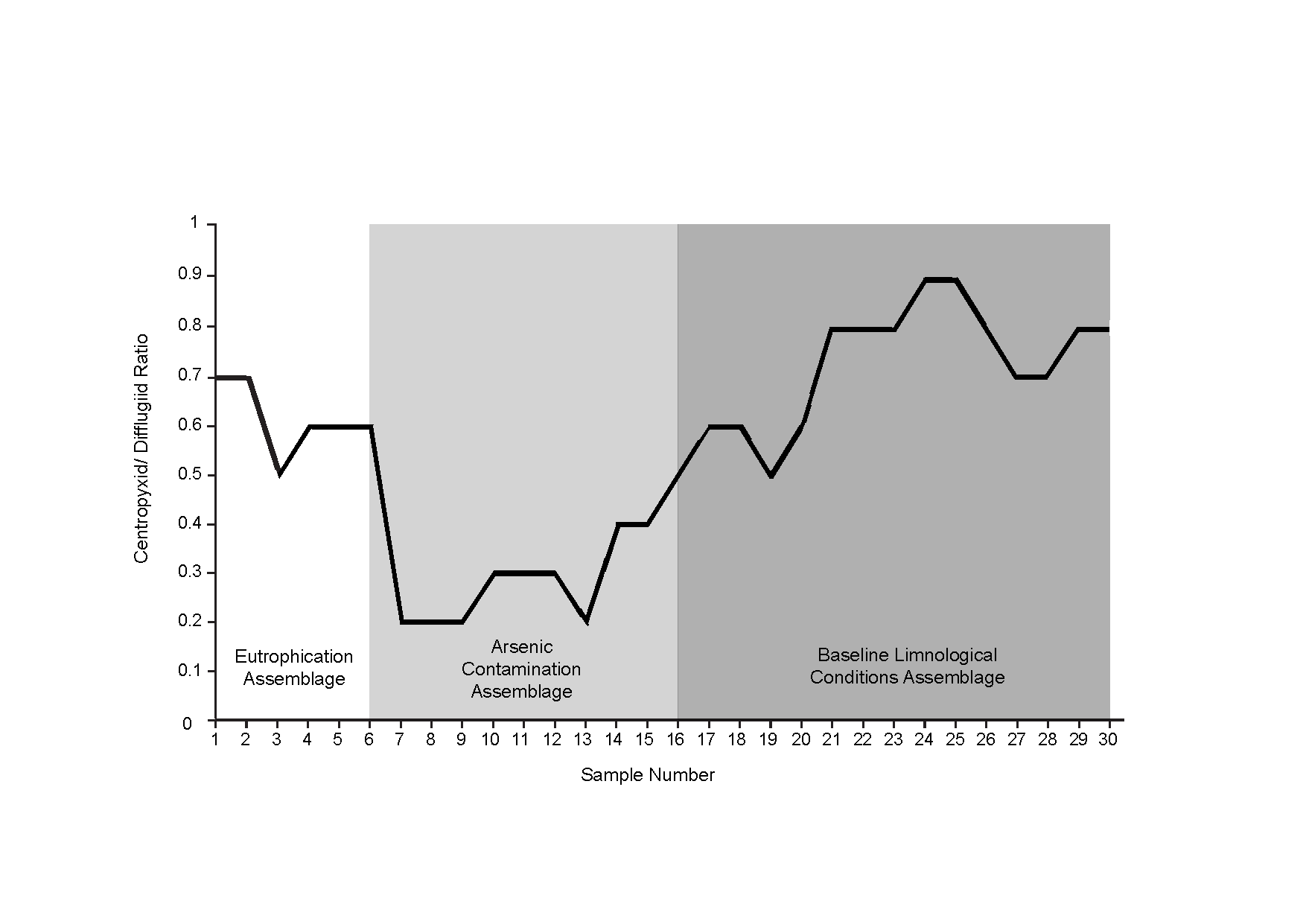

Supplement: Figure S4 [file peerj-06-4850-s004.png]
