# Supplementary material for: What killed Frame Lake? A precautionary tale for urban planners
Source: PeerJ. 2018 Jun 14;6:e4850. doi: 10.7717/peerj.4850 (PMC6004302; doi:10.7717/peerj.4850)
Supplement: Table S1 [file peerj-06-4850-s005.docx]

| **Variable** | **Spearman’s Rho** | **P-Value** | **Rank** |
| --- | --- | --- | --- |
| Minerogenics | 0.317 | 0.002 | 1 |
| Organics | 0.228 | 0.004 | 2 |
| Arsenic | 0.196 | 0.034 | 3 |
| Iron | 0.192 | 0.019 | 4 |
| Mercury | 0.168 | 0.046 | 5 |

Supplementary Table: Ranked Rho values for Spearman rank correlations with significance.
